# Assessment of therapeutic responses to gametocytocidal drugs in *Plasmodium falciparum* malaria

**DOI:** 10.1186/1475-2875-13-483

**Published:** 2014-12-09

**Authors:** Nicholas J White, Elizabeth A Ashley, Judith Recht, Michael J Delves, Andrea Ruecker, Frank M Smithuis, Alice C Eziefula, Teun Bousema, Chris Drakeley, Kesinee Chotivanich, Mallika Imwong, Sasithon Pukrittayakamee, Jetsumon Prachumsri, Cindy Chu, Chiara Andolina, Germana Bancone, Tran T Hien, Mayfong Mayxay, Walter RJ Taylor, Lorenz von Seidlein, Ric N Price, Karen I Barnes, Abdoulaye Djimdé, Feiko ter Kuile, Roly Gosling, Ingrid Chen, Mehul J Dhorda, Kasia Stepniewska, Philippe Guérin, Charles J Woodrow, Arjen M Dondorp, Nicholas PJ Day, Francois H Nosten

**Affiliations:** Mahidol Oxford Tropical Medicine Research Unit, Faculty of Tropical Medicine, Mahidol University, Bangkok, Thailand; Centre for Tropical Medicine and Global Health, Nuffield Department of Medicine, University of Oxford, Oxford, UK; Department of Life Sciences, Imperial College, London, UK; Myanmar Oxford Clinical Research Unit, Yangon, Myanmar; London School of Hygiene and Tropical Medicine, London, UK; Department of Molecular Tropical Medicine and Genetics, Faculty of Tropical Medicine, Mahidol University, Bangkok, Thailand; Department of Clinical Tropical Medicine, Faculty of Tropical Medicine, Mahidol University, Bangkok, Thailand; Mahidol Vivax Research Unit, Faculty of Tropical Medicine, Mahidol University, Bangkok, Thailand; Shoklo Malaria Research Unit, Faculty of Tropical Medicine, Mahidol University, Mae Sot, Tak, Thailand; Oxford University Clinical Research Unit, Hospital for Tropical Diseases, Ho Chi Minh City, Vietnam; Lao-Oxford-Mahosot Hospital-Wellcome Trust Research Unit, Microbiology Laboratory, Mahosot Hospital, Vientiane, Lao PDR; Global and Tropical Health Division, Menzies School of Health Research and Charles Darwin University, Darwin, NT Australia; Division of Clinical Pharmacology, Department of Medicine, University of Cape Town, Cape Town, South Africa; Malaria Research and Training Centre, Department of Epidemiology of Parasitic Diseases, Faculty of Medicine and Odonto-Stomatogy, University of Sciences, Techniques and Technologies of Bamako, Bamako, Mali; Liverpool School of Tropical Medicine, Liverpool, UK; Global Health Group, UCSF Global Health Sciences, San Francisco, CA USA; World Wide Antimalarial Resistance Network, Churchill Hospital, Oxford, Headington, UK

## Abstract

Indirect clinical measures assessing anti-malarial drug transmission-blocking activity in falciparum malaria include measurement of the duration of gametocytaemia, the rate of gametocyte clearance or the area under the gametocytaemia-time curve (AUC). These may provide useful comparative information, but they underestimate dose-response relationships for transmission-blocking activity. Following 8-aminoquinoline administration *P. falciparum* gametocytes are sterilized within hours, whereas clearance from blood takes days. Gametocytaemia AUC and clearance times are determined predominantly by the more numerous female gametocytes, which are generally less drug sensitive than the minority male gametocytes, whereas transmission-blocking activity and thus infectivity is determined by the more sensitive male forms. In choosing doses of transmission-blocking drugs there is no substitute yet for mosquito-feeding studies.

## Background

The objective of anti-malarial drug treatment is to cure the malaria infection as rapidly, reliably and safely as possible [1]. In endemic areas there is another important public health consideration and that is to stop the treated infection being transmitted to anopheline mosquitoes, and thereby to other people. Treating the asexual stage infection effectively prevents generation of young gametocytes and it also prevents recrudescences (which are associated with increased gametocyte carriage rates). This reduces the transmission potential of the treated infection [2]. *Plasmodium falciparum* differs from the other malaria parasites of humans in that the emergence of gametocytaemia is delayed with respect to asexual parasitaemia. The anti-malarial drugs which are used to treat falciparum malaria also kill young developing sexual stage parasites but they have little or no activity against the mature transmissible gametocytes. Treated infections are an important source of transmission especially in areas of low, unstable malaria transmission. Artemisinin combination treatment (ACT), the first-line treatment for falciparum malaria, reduces gametocyte carriage more than do other anti-malarial treatments [3–6], but even ACTs do not eliminate mature transmissible *P. falciparum* gametocytes [3–9]. Preventing these patients’ infections from transmitting falciparum malaria requires treatment with a specific gametocytocide, and the only generally available drug is the 8-aminoquinoline, primaquine [6–8, 10]. Primaquine rapidly sterilizes the infection, thereby reducing its transmissibility [10]. Given together with an effective treatment for the asexual stage parasites, only one dose is required. Historically this dose has been 0.5-0.75 mg base/kg [11]. Primaquine taken with food is generally well tolerated. The main adverse effect of the 8-aminoquinolines is dose-dependent oxidative haemolysis in people with glucose-6-phosphate dehydrogenase (G6PD) deficiency [10, 11]. G6PD deficiency is very common in many malaria-endemic regions. In some areas concerns over safety in patients with G6PD deficiency have limited deployment of primaquine as a gametocytocide [11].

**Figure 1:**
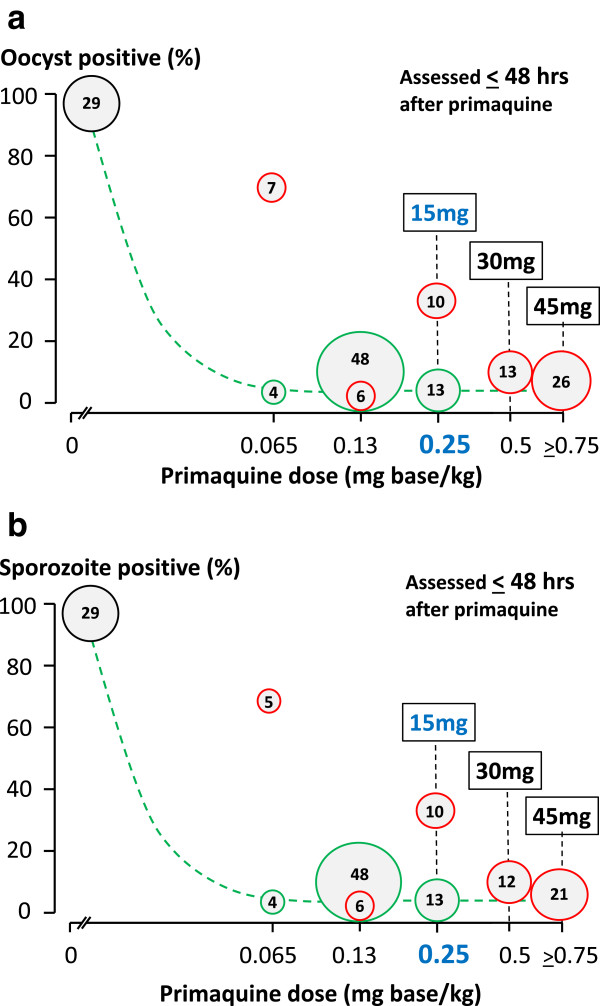
**Dose–response relationships for single-dose primaquine in reducing the infectivity of** ***Plasmodium falciparum-*** **infected subjects to anopheline mosquitoes.** Pooled data from all studies conducted [13]. Vertical axes shows the proportions of fed anopheline mosquitoes which were infected. Upper (a): Oocyst formation assessed from blood sampled 48 hours after primaquine dose; Lower (b): Sporozoite formation assessed from blood sampled 48 hours after primaquine dose. Primaquine given with an artemisinin derivative is shown in green circles, and with no anti-malarial or a non-artemisinin derivative is shown in red circles. In these studies 29 patients received no primaquine. The size of the circle is proportional to the number of subjects in each group (shown within). Corresponding adult primaquine doses are indicated in square boxes.

In efforts to contain and eliminate artemisinin-resistant falciparum malaria it is particularly important that all possible measures to reduce malaria transmission are taken. The World Health Organization (WHO) has recently recommended that the single primaquine dose for use as a *P. falciparum* gametocytocide (given together with an ACT) should be reduced to 0.25 mg base/kg as this lower dose is safer and, on the basis of the available data from direct transmission-blocking assessments from mosquito-feeding studies, was considered to be as effective as higher doses in reducing transmissibility [12–14] (Figure 1). Others have provided guidance on primaquine doses based on studies using only indirect measures reflecting *P. falciparum* gametocytaemia clearance [15]. This overview assesses the therapeutic relevance of the different measures that have been used as a basis for treatment recommendations for *P. falciparum* gametocytocides (notably primaquine).

### Dosing recommendations based on gametocytaemia; the Cochrane Review

**Figure 2:**
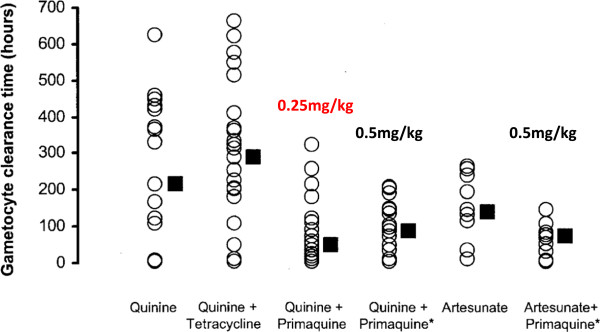
**Gametocyte clearance following different drug regimens in falciparum malaria.** Times from appearance of gametocytaemia (= 0 if gametocytaemia present on admission) to disappearance of gametocytaemia after different seven-day drug regimens in adults with acute falciparum malaria studied in Thailand [4]. Primaquine was given daily in a dose of 0.25 mg base/kg or *0.5 mg/kg.

**Figure 3:**
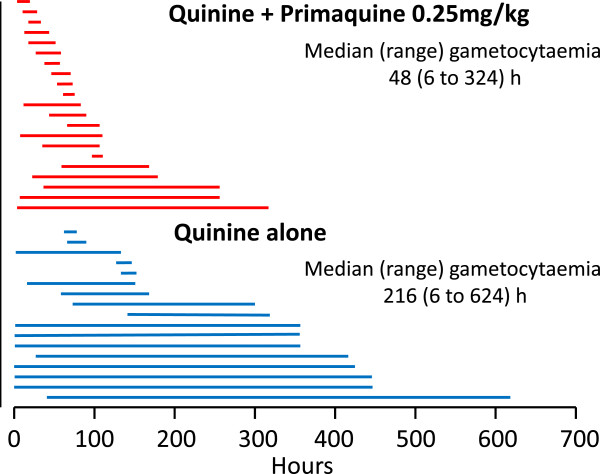
**Durations of gametocytaemia following different drug regimens in falciparum malaria.** The comparison of durations of *Plasmodium falciparum* gametocytaemia following quinine alone compared with quinine plus 0.25 mg base/kg from Pukrittayakamee *et al*. [4]. These are the same patients as in Figure 2.

A recent systematic review evaluating the role of primaquine in reducing *P. falciparum* transmission has rejected the WHO conclusions derived from the mosquito feeding data: “Current policy recommendations that 0.25 mg/kg PQ should be added as a single dose to primary treatment for *P. falciparum* malaria in areas that are targeting elimination or are facing artemisinin resistance are based on judgments and inferences rather than reliable evidence of an effect at this dose” [15]. The Cochrane Review concluded that “primaquine does reduce the duration of infectiousness” (which was equated with the period that gametocytes are detected circulating in the blood) when given at doses of 0.4 mg/kg or above (*high quality evidence*) but “for the currently recommended low dose regimen, there is little direct evidence to be confident that the effect of reduction in gametocyte prevalence is preserved” [15]. This negative conclusion was based on two studies that evaluated *P. falciparum* gametocyte clearance following primaquine given in doses less than 0.4 mg/kg. The first study was a comparative assessment of asexual and sexual parasite clearance times following seven-day regimens of quinine given either alone or together with one of the following: tetracycline, primaquine 0.25 mg base/kg, primaquine 0.5 mg/kg, or with artesunate given alone or together with primaquine 0.5 mg/kg (Figures 2 and 3) [4]. There were no significant differences in gametocyte clearance times (GCTs) between the low dose (0.25 mg/kg/d) and the high dose (0.5 mg/kg/d) primaquine-quinine regimens. The original paper [4] reported that “combinations containing primaquine resulted in significantly shorter GCTs” and concluded that “artesunate predominantly inhibits gametocyte development whereas primaquine accelerates gametocyte clearance in *P. falciparum* malaria” [4].

In contrast, the Cochrane analysis of the published study concluded that addition of primaquine 0.25 mg/kg to quinine “did not demonstrate an effect on gametocytaemia” [15]. This appears to be a mistake derived both from a mix-up between the reported effects of the lower and higher primaquine doses (median gametocyte clearance times were *shorter*, not longer, with the 0.25 mg/kg dose compared to the 0.5 mg/kg dose as shown in Figure 2), and also misreading or misinterpretation of a table describing proportions of patients who were gametocytaemic. Although the relevance of a seven-day primaquine regimen to single-dose gametocytocidal use is uncertain, the Cochrane analysis used these data to argue that a primaquine dose of 0.25 mg/kg had no effect on gametocyte clearance – which is incorrect.

**Figure 4:**
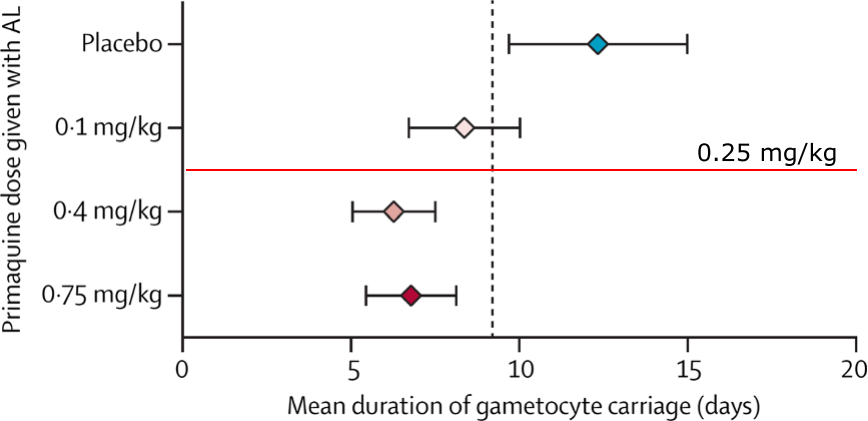
**Dose–response relationship for primaquine in reducing the duration of (female) gametocyte carriage assessed by Pfs25 transcripts.** Mean (95%CI) duration of female gametocyte carriage in Ugandan children with falciparum malaria treated with artemether-lumefantrine (AL) and different doses of primaquine as reported by Eziefula *et al.*[16]. Duration was estimated by fitting of a deterministic compartmental mathematical model to repeated *Pfs25* quantitative real time nucleic acid sequence- based analysis gametocyte prevalence estimates. The vertical dashed line indicates the set threshold for non- inferiority compared with the primaquine 0.75 mg/kg reference group (non-inferiority margin of 2.5 days). The currently recommended 0.25 mg/kg dose is indicated by the horizontal line.

The second study of low dose primaquine assessed in the Cochrane Review was a detailed recent comparison of gametocyte clearance following artemether-lumefantrine treatment of 468 Ugandan children with falciparum malaria [16]. The children were randomized to receive placebo or single primaquine doses of 0.1, 0.4 or 0.75 mg/kg. Gametocyte carriage was measured by a sensitive mRNA PCR method [16]. The results showed that in terms of gametocyte carriage reduction primaquine 0.4 mg/kg was non-inferior to 0.75 mg/kg. The gametocyte clearances following the 0.1 mg/kg dose did cross the non-inferiority margin (compared with 0.75mg/kg) and occupied an intermediate position compared with placebo (Figure 4). The Cochrane analysis concluded that “the trial evaluating low dose PQ category (0.1 mg/kg) did not demonstrate an effect” [15]. In interpreting dose-response studies (or indeed any continuous series) all data should be taken into account. By extrapolation from the observed dose-response relationship, the currently recommended 0.25 mg/kg primaquine dose would be predicted to reduce the duration of gametocyte carriage by between 50 and 100% of the maximum observed (Figure 4). Further studies are underway to assess whether this prediction is correct. Whilst the published study cannot be used to predict confidently what effect primaquine 0.25 mg/kg would have had; to use it as evidence of “no effect” is also incorrect. Aside from these misinterpretations, it is questionable whether dosage recommendations for drugs which are given to reduce malaria transmissibility should be derived primarily from studies reporting indirect measures based on gametocyte densities, such as the proportion of patients with gametocytaemia on day 8 or the area under the gametocyte time curve, as surrogates of transmission-blocking activity and thus infectivity [15].

Transmission of human malaria requires that the gametocytes are infectious to feeding anopheline mosquito vectors. Mosquito-feeding studies assess transmission-blocking effects directly and are therefore the most appropriate way to measure the pharmacodynamic effect for which the drug is prescribed. However, they are notoriously difficult, and as a consequence only few have been conducted [10]. It is much easier to measure gametocyte densities over time in blood samples either by microscopy or by molecular methods, and so there are many more data on these indirect assessments [2–5, 7–9, 16, 17]. These indirect measures based on gametocyte densities in blood have been used in drug evaluations based on the assumption that their results agree closely with transmission-blocking activity and can therefore be substituted for direct assessments. But it is well known that gametocytaemia does not equate with infectivity [10, 18]. The generally sigmoid relationship between gametocyte density and mosquito infectivity is very variable with some patients infecting mosquitoes without detectable gametocytaemia and others with high densities not being infectious.

### Gametocyte biology

**Figure 5:**
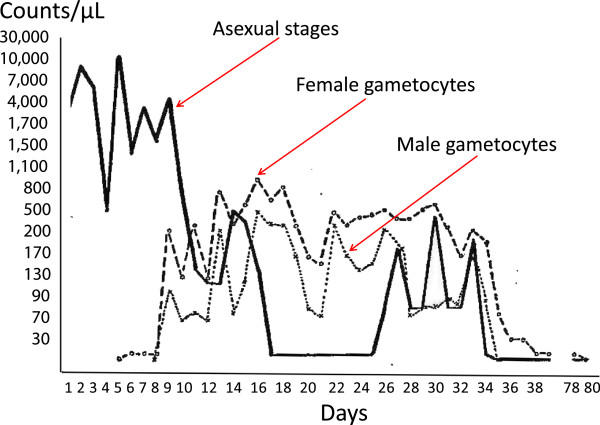
**Male and female gametocytaemia in falciparum malaria.** Artificial infection with *Plasmodium falciparum* for malariatherapy showing typical delay between the peaks of asexual and sexual parasitaemia, and the respective densities of male and female gametocytes as reported by Ciuca *et al.*[22].

Transmission of malaria requires that a feeding female anopheline vector mosquito ingests at least one mature male and one mature female gametocyte in a blood meal, and that the subsequent ookinete forms an oocyst in the mosquito’s gut wall. Once the oocyst has matured it ruptures liberating sporozoites which migrate to the salivary glands. Successful inoculation of the sporozoites transmits malaria. For some drugs (e.g., antifolates) the transmission-blocking effects are more evident in the mosquito (sporontocidal activity) which would not be reflected in indirect assessments of transmission-blocking activity from serial gametocyte counts. Malaria gametocytes arise from blood stage schizonts, which have committed to sexual stage development, so they arise from asexual parasites [18–20]. In falciparum malaria, in contrast to the other malaria infections of humans, the emergence of gametocytes is delayed [10, 18, 20] (Figure 5). Gametocytaemia peaks seven to ten days after the peak in asexual stage parasite densities [21]. The younger, more drug sensitive, sexual stages (Stages I to IV) are sequestered and then released back into the circulation as Stage V gametocytes.

The ratio of female to male gametocytes is unequal in falciparum malaria with a strong female bias in most studies [20, 23, 24]. This is presumably because each male gametocyte can generate eight microgametes and can therefore potentially fertilize eight females. There is variation in reported female to male ratios in acute malaria with values of 3-5 to 1 being observed mainly [22, 24–30] (Figure 5). At lower densities in anaemic children with chronic infections, the female to male gametocyte ratios are reportedly lower [31], but at the higher densities in acute infections in which drug assessments are performed females consistently comprise >70% of the *P. falciparum* gametocytes. The biology of male and female gametocytes is very different. Since the early days of malariatherapy it was recognized that successful transmission of malaria to mosquitoes required fully mature gametocytes, and microgametocytes which readily exflagellated. Crucially it is these minority male (micro)gametocytes which are usually more drug sensitive [32]. In 1928 Mollow described greater sensitivity of male gametocytes to the 8-aminoquinoline plasmochin (plasmoquine, pamaquine) the predecessor of primaquine [33]. Shute and Maryon in their extensive experience of artificial infection (malariatherapy) studies noted the following for *P. falciparum*: “in our cases female gametocytes almost invariably make their appearance several days before males and throughout the period of their presence the females outnumber the males from between 5 and 20 to 1 or more, and then when the gametocytes begin to diminish it is the males which fall off in numbers, and the females appear to persist for a longer period in the peripheral blood” [34]. Thus if a drug killed all the male gametocytes but none of the females, it would sterilize the infection but gametocyte density would fall by less than 30%.

### Counting gametocyte densities

Serial asexual parasite counts in blood are used to assess therapeutic responses to antimalarial drugs. Asexual parasite clearance times are dependent on several factors in addition to the drug treatment including initial parasite counts, proportion of *P. falciparum* asexual parasites which are sequestered, immunity, and the counting methods used [35]. These factors also affect gametocyte clearance assessments. However gametocyte densities are orders of magnitude lower in acute malaria so counting errors are correspondingly greater. Gametocyte densities are usually quantitated on thick blood films with males and females counted together. There is input to the circulation from sequestered immature gametocytes while dead gametocytes are cleared predominantly by the spleen, so in drug assessments gametocyte input needs to be deconvoluted from clearance. Artemisinins, and to a lesser extent other anti-malarials which act on blood stage parasites, mainly reduce the input into the circulation whereas primaquine (and other 8-aminoquinolines) accelerate clearance [4]. Live gametocytes may circulate for many days (mean estimates 4.5 to 6.5 days) [36]. Microscopy cannot distinguish reliably between younger Stage V gametocytes which have just entered the circulation and are not yet infectious and older Stage V gametocytes which are infectious. This explains why gametocytaemic patients with acute malaria may not infect mosquitoes when first assessed. Microscopy also cannot distinguish between live and dead gametocytes. Assessment of the sex ratio by microscopy requires experience, and is more difficult on thick films and after treatment with gametocytocidal drugs. Measurement of mRNA transcripts by qPCR methods is more sensitive than microscopy. The most widely used is the QT-NASBA (quantitative nucleic acid sequence-based amplification) based on detection of Pfs25 transcripts [8, 16, 36–38]. Importantly Pfs25, and its orthologues in rodent malarias, are expressed much more abundantly in female gametocytes. Indeed they appear to be female specific (although whether Pfs 25 is transcribed but not expressed in male gametocytes still needs to be determined) [32, 38, 39].

### Gametocyte clearance assessment

**Figure 6:**
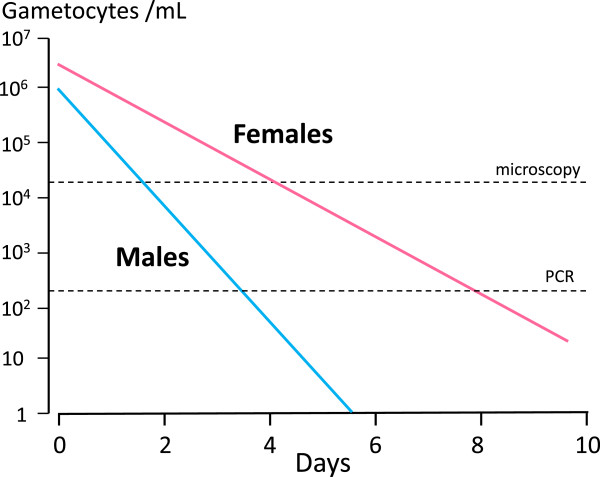
**Proposed relationship between male and female gametocyte clearance and transmission blocking effects in falciparum malaria.** If male gametocytes are more sensitive to transmission-blocking drugs than female gametocytes, and female gametocytes predominate, then gametocyte clearance times are determined by the female gametocytaemia, and transmission-blocking effects are determined mainly by male gametocytaemia. In this illustration of gametocytaemia responses to drug treatment clearance half-lives are one day for male (blue) and two days for female gametocytes (pink). The limits of gametocyte detection by microscopy and Pfs25mRNA (which detects predominantly females) are shown by the dotted lines. If a density of >1,000/mL was required for mosquito infection, then in this illustration the maximum duration of possible infectivity is three days compared to a clearance time measured by Pfs25mRNA of seven days. In fact, drugs such as primaquine sterilize rapidly (Figure 7) suggesting that loss of infectivity precedes gametocyte clearance so the post-treatment duration of infectivity in this illustration is likely to be < one day.

**Figure 7:**
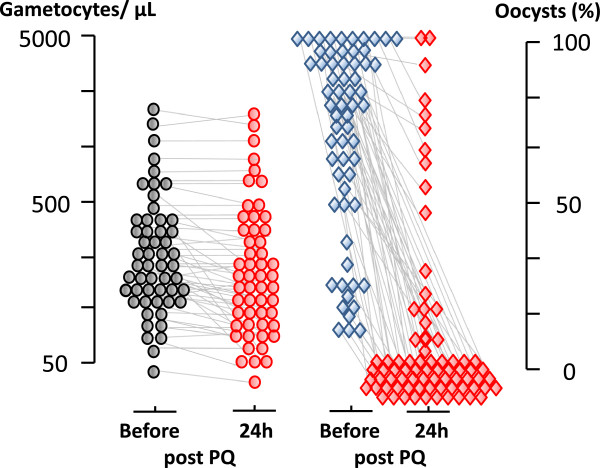
**The temporal dissociation in falciparum malaria between changes in gametocytaemia and effects on infectivity following primaquine administration.** Individual data from studies of mosquito infectivity following anti-malarial treatments of falciparum malaria with plasmoquine or primaquine in which oocyst assessments were made in mosquitoes which fed ~24 hours after drug administration [10]. Oocysts were assessed typically in 10-20 mosquitoes 6-7 days after feeding. Each pair of circles or diamonds represents a studied patient.

The limited available evidence suggests that gametocyte clearance is a first order process. Ideally in drug comparisons or dose-response assessments this would be assessed by a first order rate constant or half-life, as for asexual parasite clearance [35], but gametocyte counts have seldom been taken frequently enough for this. Instead the number of days of detectable gametocytaemia, proportions of patients with patent gametocytaemia on specific days (particularly day 7 or day 8), and the area under the gametocyte time curve (AUC) have been reported [3, 4, 7–9, 15–17, 27, 28, 36, 40–42]. These individual values are usually pooled for the whole treated patient groups to account for the majority of patients who do not have patent gametocytaemia, in order to provide an estimate of the overall treated population’s gametocyte carriage at potentially transmissible densities. There are several important considerations and limitations to these measures of gametocytocidal drug effects based on gametocyte density estimates:

1. Gametocyte clearance time estimates depend on the detection methods used. Times derived from the more sensitive QT-NASBA are obviously longer than those based on microscopy. Times derived from thick film counts per 1000 white cells will be longer than those per 200 white cells. Fitting a mathematical model of gametocyte dynamics to measurements of sub-microscopic gametocyte prevalence and density [36] to estimate of time to gametocyte clearance has also been used as a measure of drug effect [16]. As for asexual parasite clearance estimates, the times to clearance of gametocytaemia are proportional to initial gametocyte densities and the quality and sensitivity of microscopy or the assay used. Whatever method is used the higher the initial density the longer it takes before densities fall below the level of detection [35] (Figure 6).
2. Male gametocytes are usually more drug sensitive than females [32], and it is very likely therefore that they are cleared more rapidly than female gametocytes [33, 34] (Figure 6). Total gametocyte counts by microscopy reflect the predominant female gametocyte population (~70-90% of the total) – and Pfs25 QT-NASBA provides estimates predominantly or only for female gametocytes. If the male gametocytes are more drug sensitive than the females, and they are cleared more rapidly, then the gametocytaemia AUC is determined largely, and the proportions of patients with gametocytaemia on day 8 and the gametocyte clearance times are determined *solely* by the less drug-sensitive females (see Figure 6).
3. It is not known how rapidly dead gametocytes are cleared from the blood.
4. The relationship between *P. falciparum* gametocyte density in blood and infectivity to mosquitoes is very variable, and is also time dependent (see below) [10, 18].
5. The area under the gametocytaemia time curve (AUC), whilst providing a model independent measure, assumes that density and time are equally important in terms of infectivity to mosquitoes, which clearly they are not. Without logarithmic transformation a gametocyte density of 1,000/uL for three days provides the same AUC as a density of 100/uL for 30 days. With logarithmic transformation this becomes 4.5 days. High gametocyte densities in patients presenting with acute symptomatic falciparum malaria are often not infectious at all, comprising the first wave of young Stage V gametocytes, whereas low densities later on are usually infectious [18, 34, 43]. In the example above gametocyte carriage at transmissible densities for 30 days provides ten times more opportunity for mosquitoes to bite than carriage for three days.
6. 8-aminoquinolines sterilize malaria infections within hours, but gametocyte clearance does not start to increase for > one day [10] (Figure 7), yet the first day post-treatment may contribute a significant proportion of the total AUC.
7. Use of the AUC also assumes a linear relationship between gametocyte density and infectivity whereas in reality this relationship is sigmoid [10, 18, 43, 44]. Unusual patients with very high gametocyte densities contribute disproportionately to the AUC estimate, although their potential infectivity is saturated.

### Cochrane review analysis of gametocytaemia in drug comparisons; the AUC

The Cochrane review [15] used the following formulas for the area under the gametocyte –time curve.

AUC (days 1 to 15) = ((8-1)*(G1 + G8)/2) + ((15-8)*(G15 + G8)/2)/14 for days 1 through 15.

AUC (days 1 to 29) = ((8-1)*(G1 + G8)/2) + ((15-8)*(G15 + G8)/2) + ((29-15)*(G29 + G15)/2)/28 for days 1 through 29.

AUC (days 1 to 43) = ((8-1)*(G1 + G8)/2) + ((15-8)*(G15 + G8)/2) + ((29-15)*(G29 + G15)/2) + ((43-29)*(G43 + G29)/2)/42 for days 1 through 43

where Gx = mean gametocyte density on day X (estimated using data from all participants still enrolled on day X). The log(10) AUC values were estimated using geometric mean gametocyte density.

### Infectivity

**Figure 8:**
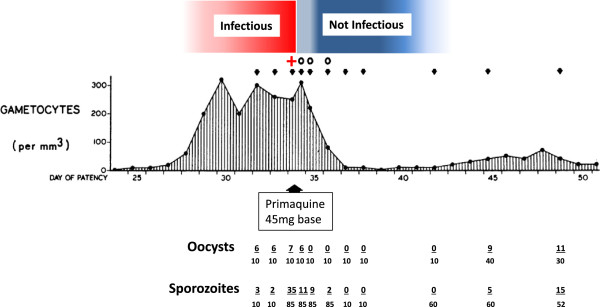
**The effects of primaquine alone on asexual and sexual parasitaemia in falciparum malaria.** Volunteer study reported by Rieckmann *et al*. [52] in which primaquine (45 mg base) only was given. The volunteers were infected with the Malayan Camp strain of *P. falciparum. Anopheles stephensi* was used as the vector. Volunteer 3 data are shown. Gametocytaemia was quantitated by microscopy, oocysts and sporozoites were assessed by microscopy after dissection (arrows are the days when mosquitoes were dissected, the numerator is the number positive, the denominator is the number of mosquitoes dissected). The subsequent infectivity of these mosquitoes (i.e., from the same batches) to other volunteers was also assessed; a different, healthy, non-immune volunteer was bitten by 75 mosquitoes before, and 12, 24 and 48 hours after primaquine had been given to volunteer 3; + (red) denotes that the volunteer became infected with falciparum malaria, 0 denotes the volunteer did not become infected.

**Figure 9:**
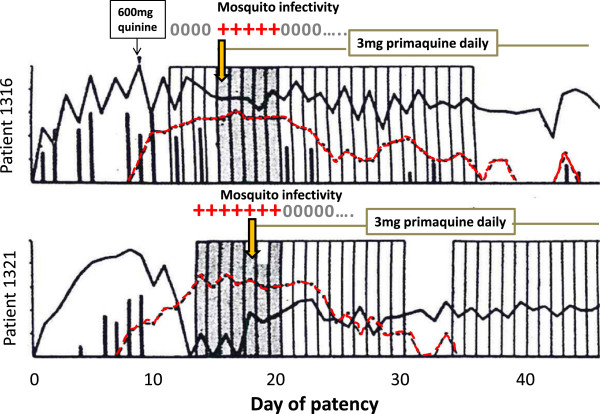
**Effects of very low dose primaquine on transmissibility of falciparum malaria.** Malariatherapy with *Plasmodium falciparum* (Panama strain) in two patients who were treated with primaquine 3 mg daily (starting at vertical yellow arrow) as reported by Young *et al*[53]. Daily direct mosquito feeding was performed. Black line is asexual parasitaemia, vertical lines are temperature (fever), vertical boxes represent mosquito feeds and dark hatching shows proportion infected. Mosquitoes (10 each) were dissected for oocysts and sporozoites; red + = infected (≥8 of 10), grey 0 = none infected. Note that infectivity persisted until the fourth day of treatment in patient 1316 (upper panel), and the second day in patient 1321 (lower panel) and also that the young circulating gametocytes in patient 1316 were initially not infectious. There was a moderate and delayed effect on gametocytaemia (red dashed line).

Assessment of the reduction in infectivity of gametocytes to female anopheline mosquitoes by direct or membrane feeding measures the pharmacological effect that is intended [45–49]. Direct feeding is more clinically relevant, but may be deemed unacceptable by ethics review boards. Although there may be absolute differences between the two methods, relative differences such as the relationships between dose, drug exposure and transmission-blocking effects should be similar. Batches of the fed mosquitoes are examined for gut oocysts and later for salivary gland sporozoites to capture any additional pharmacological effects on parasite development in the mosquito. Mosquito-feeding studies are difficult, laborious and expensive – and therefore seldom done. Such studies may underestimate transmission-blocking effects at lower gametocyte densities (which are more common because of the geometric distribution of gametocytes in human populations) [10]. In the reported artificial malaria infection experiments the subjects commonly had high gametocyte densities for several days, which were at the upper end of the distribution of gametocyte densities encountered in natural infections, and they were highly infectious (Figures 5, 8 and 9). This resulted in heavy and potentially detrimental mosquito infections (often >30 oocysts per gut). Drug effects on gametocyte viability are continuous, but the final effect on transmission to a mosquito is approximately binary – it is either infected or not, and only one successful oocyst is all that is necessary for its potential infectivity. For example, aside from any sterilizing activity, if there were 1,000 viable gametocytes/uL of blood, a 99% reduction would still leave 10 gametocytes/uL - which may still be infectious; whereas if there were 10 gametocytes/uL initially, then a 99% reduction leaves 0.1/uL, which is unlikely to infect [10]. This contrasts with natural conditions where gametocyte densities in blood are generally much lower than in the volunteer studies. Indeed the majority of densities are below the limits of microscopy detection [44, 50]. Infected wild anopheline mosquito vectors when examined have correspondingly less intense infections (median 2 oocysts per gut) [51]. Thus artificial infection studies tend to underestimate transmission-blocking drug effects at a population level.

Comparison of changes in gametocytaemia and infectivity reveals important differences. Whereas 8-aminoquinolines sterilize falciparum malaria infections rapidly, usually within hours of exposure, gametocyte densities in blood do not decline for at least one day [10]. This suggests that at least the first day’s AUC should be discarded from AUC comparisons involving 8-aminoquinolines. There are several possible explanations for the significant differences between gametocytaemia clearance and reduction in infectivity:

1. Gametocyte sterilization is rapid but killing is slower.
2. Clearance of dead gametocytes from the blood is slow.
3. 8-aminoquinolines affect predominantly the minority male gametocyte population [32].

**Figure 10:**
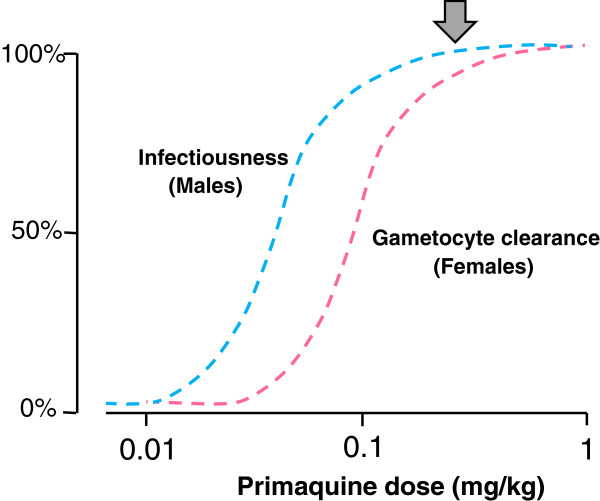
**Proposed approximate dose-response relationships for primaquine in falciparum malaria.** Approximate dose-response relationships in falciparum malaria for primaquine inhibition of infectiousness (blue dashed curve) presumably reflecting susceptibility of male gametocytes, and effects on gametocyte clearance (pink dashed curve) reflecting susceptibility of female gametocytes. Arrow shows currently recommended transmission-blocking primaquine dose of 0.25 mg/kg. Studies are in progress to characterize these relationships more accurately.

Clearly if drug effects are directed predominantly against a minority male gametocyte population [32] then dose-response assessments based on microscopy, which does not distinguish between the gametocyte sexes, or mRNA transcripts produced predominantly or exclusively by female gametocytes, will substantially underestimate transmission-blocking activity. The shape of the derived dose-response relationship may be correct but use of indirect measures will shift curves to the right, effectively underestimating transmission-blocking activity (Figure 10). The dose-response relationship derived from counts in infections where females predominate, or from Pfs25 transcripts, is mainly that of female gametocyte susceptibility, whereas the dose-response relationship derived from infectivity to mosquitoes is that of the more sensitive sexual stage, i.e., the male gametocytes. The primaquine dose-response relationships (given together with artemether-lumefantrine) derived from female gametocyte clearance (assessed by Pfs25 mRNA) suggest that maximum effects on gametocytaemia occurred at doses down to 0.4 mg/kg, with approximately half maximum effects at 0.1 mg/kg [15] (Figure 4). Direct estimates of infectivity inhibition from heterogeneous pooled data obtained from mosquito-feeding studies suggests maximum effects down to 0.13 mg/kg (Figures 1 and 10) [12]. More data will be needed to refine both these estimates, and to characterize male and female gametocytaemia clearance independently, but this comparison does suggest significant underestimation of the primaquine transmission blocking dose response relationship by indirect measures based upon gametocytaemia.

Although sub-microscopic densities of gametocytes are an important source of malaria transmission [7, 44, 50], this is usually from asymptomatic individuals at quasi-steady state parasitaemias. In symptomatic patients it seems reasonable to predict that soon after gametocyte densities fall below the level of microscopy detection the treated patient becomes non-infectious – provided that the asexual infection is cured (i.e., that there is not continued low-level asexual stage multiplication generating new gametocytes), and there is no re-infection. If one drug clears gametocytaemia more rapidly than another, then it seems reasonable to conclude that it will also reduce transmissibility more. More information is needed on these points. However for the reasons given above determining the dose-response relationship for a drug (such as primaquine) based on indirect measures is likely to provide a significant underestimate of the relationship for transmission-blocking activity.

### 8-aminoquinoline effects on infectivity to mosquitoes

In 1927, shortly after its development, the 8-aminoquinoline plasmoquine (the predecessor of primaquine) was shown to clear gametocytaemia more rapidly than asexual stages in falciparum malaria [54]. By 1929, studies conducted in Panama showed that plasmoquine rapidly reduced the infectivity of *P. falciparum* to anopheline mosquitoes [55, 56]. The transmission-blocking effect (assessed by reduction in mosquito oocyst counts) preceded the effects on gametocytaemia. In subsequent mosquito-feeding studies plasmoquine doses as low as 10 mg provided rapid and potent transmission-blocking activity [53, 57–60]. Primaquine replaced plasmoquine 65 years ago because it was safer and more potent; it was three times more active against pre-erythrocytic stages, four to six times better in radical curative activity against vivax malaria, and was half as toxic. The transmission-blocking activities of the two drugs were never compared but, given their structural and pharmacological similarity, it seems very likely that the pharmacodynamic effects would also be similar and very unlikely primaquine would be less active than plasmoquine. In 1959, Young reported the effects of an adult primaquine dose of 3 mg per day (0.05 mg/kg) in two malariatherapy patients. This very small dose abolished *P. falciparum* infectivity to mosquitoes by the third and fifth days of treatment, which was before any decline in gametocytaemia [53] (Figure 9). A recent review found data from 158 individual gametocytaemic subjects from different studies in which infectivity in mosquito-feeding studies was assessed both by oocyst and sporozoite production 24 and 48 hours after drug exposures [13]. Of these, 31 subjects received plasmoquine (before 1950) and 127 received primaquine (69 together with an artemisinin derivative). These studies show clearly that both plasmoquine and primaquine rapidly and potently reduce the infectivity of *P. falciparum* within hours (Figure 1), whereas there is a lag phase >24 hours before gametocyte clearance starts (Figure 7) [10].

## Discussion

Assessment of anti-malarial drug transmission-blocking activity *in vivo* is problematic. Mosquito-feeding studies assess directly the effect for which the drug is prescribed (i.e., to block transmission of malaria) but they are difficult and laborious, and there are few data as a consequence. Fortunately there is renewed interest in these assessments and more information to guide evaluations of transmission-blocking effects drugs and vaccines will soon become available. Meanwhile indirect measures based on quantitation of gametocytaemia provide a guide to the comparative activity of different drugs or different drug dosages - but they make a number of assumptions. Critically current gametocyte clearance measures probably underestimate significantly the inhibitory effects of 8-aminoquinolines on infectivity. This compromises dose-response assessments both for the time of onset and the magnitude of transmission-blocking activity. The unique studies of Rieckmann *et al.* in which volunteers were infected with *P. falciparum* and managed to a steady state of parasitaemia highlights the discrepancies between total gametocytaemia, mosquito oocyst development, sporozoite formation, and the infectivity of these mosquitoes to naïve volunteers [52, 61] (Figure 8). Early observations on the greater sensitivity of male gametocytes to plasmoquine (pamaquine) [33], the more rapid clearance of male gametocytes [34], and the recent study showing large differences between the drug susceptibility of male and female gametocytes [32] provide further reason to be cautious in interpreting drug comparisons based on total gametocytaemia quantitated by microscopy or predominantly female gametocytaemia assessed by quantitation of Pfs25 transcripts. The degree of underestimation by indirect assessments is unclear as there have been few simultaneous comparisons. A comparison of different studies (albeit with different patient groups, designs, drug dosages, etc.) in *P. falciparum* infections (summarized in Figures 1 and 7) suggests a significant difference. If infectivity is determined by the susceptibility of male gametocytes, then this comparison would suggest that male gametocytes may be more than three times more susceptible to primaquine than females (Figures 6 and 10) but further studies are required to determine whether these predictions are correct. On the other hand, it seems very unlikely that these indirect assessments based on gametocyte clearance *overestimate* transmission-blocking activity so they do set an upper limit for a dose-response relationship which may be useful.

More direct assessments of drug effects on male and female gametocytes individually such as the *P. falciparum* dual gamete formation assay (*Pf*DGFA) are an advance [62, 63], and histochemical methods may distinguish live from dead gametocytes, but for the present for dose-response assessments there is no substitute for mosquito-feeding studies (although close attention must be given to the specific design of these assays to ensure that the outcomes can be interpreted and compared usefully) [45–49]. Primaquine is the only generally available drug, which rapidly and reliably reduces the transmissibility of *P. falciparum* malaria infections from patients presenting with patent gametocytaemia. Unfortunately the 8-aminoquinolines require hepatic bioactivation which compromises *ex vivo* assessments. Characterizing sex-specific markers and assessing the pharmacokinetic-pharmacodynamic relationship for primaquine’s gametocytocidal activity on male and female gametocytes *in vivo* is a research priority. Ideally dose-response assessments should be performed by comparing the infectiousness of gametocytaemic blood to anopheline mosquitoes before and after different doses, preferably with assessment of both oocyst and sporozoite formation, plasma primaquine concentration measurement (and CYP2D6 genotyping) [64]. Given the pharmacokinetic properties and the documented rapidity of onset (particularly for inhibition of sporozoite formation) testing before and eight to 24 hours after dosing would reduce variability in the feeding mosquitoes.

## Conclusion

Despite their difficulty, mosquito-feeding studies still remain the ‘gold standard’ for the assessment of transmission-blocking dose-response relationships. More information is needed on the relative susceptibility of male and female gametocytes and the kinetics of male and female gametocyte killing, together with direct comparisons with infectivity assessments, before indirect measures can be used confidently to make dosage recommendations.
